# Relationship Between Brain Pulsatility and Cerebral Perfusion Pressure: Replicated Validation Using Different Drivers of CPP Change

**DOI:** 10.1007/s12028-017-0404-9

**Published:** 2017-05-25

**Authors:** Leanne A. Calviello, Nicolás de Riva, Joseph Donnelly, Marek Czosnyka, Peter Smielewski, David K. Menon, Frederick A. Zeiler

**Affiliations:** 1 Division of Neurosurgery, Department of Clinical Neurosciences, Addenbrooke’s Hospital, University of Cambridge, Cambridge, UK; 2 Division of Neuroanesthesia, Department of Anesthesiology, Hospital Clinic, Universitat de Barcelona, Barcelona, Spain; 3 Division of Anesthetics, Addenbrooke’s Hospital, University of Cambridge, Cambridge, UK; 4 Section of Neurosurgery, Department of Surgery, Rady Faculty of Health Sciences, University of Manitoba, Winnipeg, Canada; 5 Clinician Investigator Program, University of Manitoba, Winnipeg, Canada; 6 Neurosciences Critical Care Unit, Addenbrooke’s Hospital, Cambridge, UK; 7 Queens’ College, Cambridge, UK; 8 National Institute for Health Research, Cambridge, UK

**Keywords:** Neurocritical care, Traumatic brain injury, Pulsatility index, Intracranial pressure, Cerebral perfusion pressure, Transcranial doppler

## Abstract

**Background:**

Determination of relationships between transcranial Doppler (TCD)-based spectral pulsatility index (sPI) and pulse amplitude (AMP) of intracranial pressure (ICP) in 2 groups of severe traumatic brain injury (TBI) patients (a) displaying plateau waves and (b) with unstable mean arterial pressure (MAP).

**Methods:**

We retrospectively reviewed patients with severe TBI and continuous TCD monitoring displaying either plateau waves or unstable MAP from 1992 to 1998. We utilized linear and nonlinear regression techniques to describe both cohorts: cerebral perfusion pressure (CPP) versus AMP, CPP versus sPI, mean ICP versus ICP AMP, mean ICP versus sPI, and AMP versus sPI.

**Results:**

Nonlinear regression techniques were employed to analyze the relationships with CPP. In plateau wave and unstable MAP patients, CPP versus sPI displayed an inverse nonlinear relationship (*R* ^2^ = 0.820 vs. *R* ^2^ = 0.610, respectively), with the CPP versus sPI relationship best modeled by the following function in both cases: PI = *a* + (*b*/CPP). Similarly, in both groups, CPP versus AMP displayed an inverse nonlinear relationship (*R* ^2^ = 0.610 vs. *R* ^2^ = 0.360, respectively). Positive linear correlations were displayed in both the plateau wave and unstable MAP cohorts between: ICP versus AMP, ICP versus sPI, AMP versus sPI.

**Conclusions:**

There is an inverse relationship through nonlinear regression between CPP versus AMP and CPP versus sPI display. This provides evidence to support a previously-proposed model of TCD pulsatility index. ICP shows a positive linear correlation with AMP and sPI, which is also established between AMP and sPI.

**Electronic supplementary material:**

The online version of this article (doi:10.1007/s12028-017-0404-9) contains supplementary material, which is available to authorized users.

## Introduction

Multi-modal, high-resolution intracranial monitoring within the critically-ill neurological patient is becoming standard in most high-volume neurocritical care units. Recent endorsement of multi-modal monitoring has come from a multitude of professional societies associated with the critical care management of these patients [1, 2]. To date, traumatic brain injury (TBI) and subarachnoid hemorrhage have dominated the literature on both invasive and noninvasive cranial monitoring, with TBI the focus of most publications [1, 2].

Worldwide interest in noninvasive measurement of various cranial hemodynamic indices has driven the application of transcranial Doppler (TCD) in a variety of scenarios, with the goal of correlating middle cerebral artery (MCA) flow velocity and pulsatility index (PI) to common invasive measures such as intracranial pressure (ICP) and cerebral perfusion pressure (CPP, the calculated difference between arterial blood pressure (ABP) and ICP), as documented within a recent systematic review [3]. The brain is extraordinarily fragile following TBI. Patients are at risk of increasing ICP, and of sudden changes in ABP or CPP that may require immediate clinical intervention. Low CPP is associated with potential instances of delayed cerebral ischemia; conversely, high CPP is associated with edema [4].

PI has been found to be a complex descriptor of several “mutually interdependent” parameters within the brain [4]. Elevated values of PI can signal rising ICP and can additionally inform of both decreasing CPP and of decreasing cerebrovascular resistance. These correlations are particularly relevant to the study of plateau waves, phenomena characterized by unexpected elevations in ICP above 50 mm Hg accompanied by marked depletions of CPP for a duration of at least 5 min that either resolve on their own or through treatment with vasopressors. In addition to plateau waves, alterations of mean arterial pressure (MAP) can upset the balance of CPP in critically-ill neurological patients, due to the fundamental nature of ABP within the CPP derivation. Clinical analysis of unstable, decreasing MAP can assist in the ongoing investigation of the relationships between various cerebral hemodynamic parameters.

To delineate the relationships between CPP, ICP, MAP, and TCD parameters, continuous data series through large ranges of CPP and ICP values would be ideal. Difficulties with long-term, high-quality TCD signal acquisition have led to limited studies in humans correlating TCD measures to CPP, ICP, and MAP [5], with some animal studies documenting the relationship [6] and others utilizing mathematical modeling [7]. Ideally, being able to correlate TCD-based PI with ICP pulse amplitude (AMP), MAP, and CPP could bolster the concept of reliable noninvasive measurement of these hemodynamic parameters. Previous literature has outlined the possibility of an inverse nonlinear correlation between PI and CPP, utilizing “spectral” PI (sPI, defined as the first harmonic of the flow velocity (FV) pulse waveform divided by mean FV) in 51 patients with plateau waves and continuous TCD monitoring [6]. The following relationship between PI and CPP was proposed within the supplementary portion of that same manuscript [4]:

$${\text{PI}} = \frac{{A_{1} }}{{{\text{CPP}}m}} \cdot \sqrt {\left( {{\text{CVR}} \cdot {\text{Ca}}} \right)^{2 } {\text{HR}}^{2} \cdot \left( {2\pi } \right)^{2} + 1}$$

In this equation, *A*_1_ represents the fundamental harmonic of ABP, CPPm the calculated mean of recorded CPP values, CVR the cerebrovascular resistance, Ca the cerebral arterial compliance, and HR the heart rate.

Given the complexities in such analyses, we hypothesized that validation of relationships between CPP and indices of cerebrovascular pulsatility (defined using either sPI or AMP) would be strengthened by demonstrating similar relationships in contexts where the drivers of CPP change were different. Consequently, in this study, we used a unified method to compare the same relationship in clinical conditions where CPP is affected either by increasing ICP or by the oscillations of unstable MAP. The aim of our study was to describe and compare the relationships between spectral PI and various invasively-derived cerebral hemodynamic measures across two groups of TBI patients demonstrating either plateau waves or unstable MAP while continuously recording flow velocities with TCD. These patients were of interest given the continuous data recorded through a wide range of CPP values, allowing us to potentially gain a better insight into the relationship between TCD and invasively-monitored parameters. The following relationships are described for each cohort: ICP versus AMP, ICP versus sPI, AMP versus sPI, CPP versus AMP, and CPP versus sPI.

## Methods

### Patients

From a database of 1023 head-injured patients with continuous ICM+ (Intensive Care Monitoring) monitoring and TCD recordings of ABP and ICP, we performed a retrospective review of recorded data for patients exhibiting ICP plateau waves during the period from 1992 to 1998. We were primarily observing physiological effects in subsets of TBI patients, with plateau waves of special interest because they are relatively uncommon. Each recording lasted for a maximum of 15–30 min. These patients have previously been described within other published studies [6, 8, 9] and were selected to evaluate the relationship between CPP versus sPI and CPP versus AMP over a large range of CPP that was observed secondary to large fluctuations in ICP, as seen during plateau waves. 5643 minute-by-minute data points for each variable were analyzed across all patients.

Furthermore, we retrospectively analyzed a second cohort of severe TBI patients with unstable MAP to determine the relationship between CPP versus sPI and CPP versus AMP during wide fluctuations in CPP secondary to unstable MAP. The definition of “unstable MAP” describes mean ABP during recording changing by a minimum of 15 mm Hg in either a monotonic or a fluctuating manner. All patients in both cohorts suffered moderate–severe TBI and were admitted to the Neurosciences Critical Care Unit (NCCU) at Addenbrooke’s Hospital, Cambridge. Patients were managed according to an ICP-oriented protocol which aimed to keep ICP below 20 mm Hg. Institutional ICP protocols were employed during the patients’ NCCU stay, to provide homogeneity of care between patients. Of note, these patients were not treated via CPP-directed therapies, as this was not the standard of care within the NCCU at that time. Thus, fluctuations in CPP seen during plateau wave recordings are natural CPP responses, with no influence of vasoactive substances during recording. On the other hand, patients within the unstable MAP cohort may have received vasopressors in an attempt to stabilize blood pressure; however, this was not titrated to CPP goals.

### Monitoring

All patients underwent both invasive and noninvasive monitoring throughout their ICU stay. Raw data signals from select monitoring devices were recorded and electronically stored using WREC software (Warsaw University of Technology).

ABP was continuously monitored both invasively (from the radial artery using a pressure monitoring kit [Baxter Healthcare CA, USA; Sidcup, UK]) and noninvasively. ICP was monitored using an intraparenchymal probe with strain gauge sensors (Codman & Shurtleff, MA, USA, or Camino Laboratories, CA, USA). Mean and peak blood flow velocities (FVm and FVx, respectively) were monitored from the MCA with a 2 MHz probe.

Raw data recordings within the plateau wave cohort patients included only 20–40 min of continuous data, focusing on the immediate periods before, during, and after ICP plateau waves. Within the unstable MAP cohort, raw data recording occurred throughout the entire period of unstable blood pressures.

Monitoring of above brain modalities was conducted as a part of standard NCCU patient care using an anonymized database of physiological monitoring variables in neurocritical care. Data on age, injury severity, and clinical status at hospital discharge were recorded at the time of monitoring on this database, and no attempt was made to re-access clinical records for additional information. Since all data were extracted from the hospital records and fully anonymized, no data on long-term outcomes or patient identifiers were available, and formal patient or proxy consent was not sought.

### Data Processing

Processing of raw data signals utilized ICM + software (Cambridge Enterprise, Cambridge, UK; http://www.neurosurg.cam.ac.uk/icmplus). Signal artifact removal was first conducted with signal cropping tools within ICM+. CPP was determined from the difference between raw ABP and ICP signals.

Primary analysis involved the calculation of time-averaged mean values for ABP (MAP), ICP, cerebral blood FV, and CPP. These means were calculated during 10-s time windows and were updated every 10 s to eliminate overlap. Mean FV was calculated using the data from FV. In addition, we determined the amplitude of the fundamental frequency of FV (F1) and the amplitude of the fundamental frequency of ICP AMP. Both fundamental amplitude calculations were done by applying a 20-sec time window, updated every 10 sec.

Final data processing involved the calculations of sPI over the course of each individual recording utilizing the equation: Mean F1/Mean FV. Mean F1 and FV were calculated utilizing a 10-sec time window, updated every 10 sec.

All data post-processing was exported from each patient to separate comma-separated variable (CSV) files for further statistical analysis.

### Statistics

All statistical analyses were conducted utilizing the XLSTAT (Addinsoft, New York, USA; https://www.xlstat.com/en/) add-on package to Microsoft Excel (Microsoft Office 15, Version 16.0.7369.1323) and IBM SPSS Statistics 23 software. Post-processing data of individual patients, as CSV documents, were compiled into one CSV document containing all patients and signals described previously. Statistical significance for measured and derived variables, both within and between the two patient cohorts, was determined utilizing a two-tailed *t* test, with an alpha set at 0.05.

Various statistical techniques were employed to describe the following relationships in both patient cohorts: ICP versus AMP, ICP versus sPI, AMP versus sPI, CPP versus AMP, and CPP versus sPI.

Relationships between ICP, AMP, and sPI were analyzed utilizing linear regression techniques. Goodness of fit was reported utilizing the Pearson correlation coefficient (*r*) and the determination coefficient (*R* ^2^). All *R* ^2^ values were reported. Statistical significance was assigned only if the *p* value was less than 0.05.

Analysis of the relationship between CPP, AMP, and sPI was conducted utilizing both linear and nonlinear techniques, with goodness of fit reported via *R* ^2^. Nonlinear regression involved the fitting of existing functions within the statistical programs, in addition to manual function fitting utilizing the nonlinear inverse function: *y* = *a* + (*b*/*x*).

## Results

### Patient Demographics

**Fig. 1:**
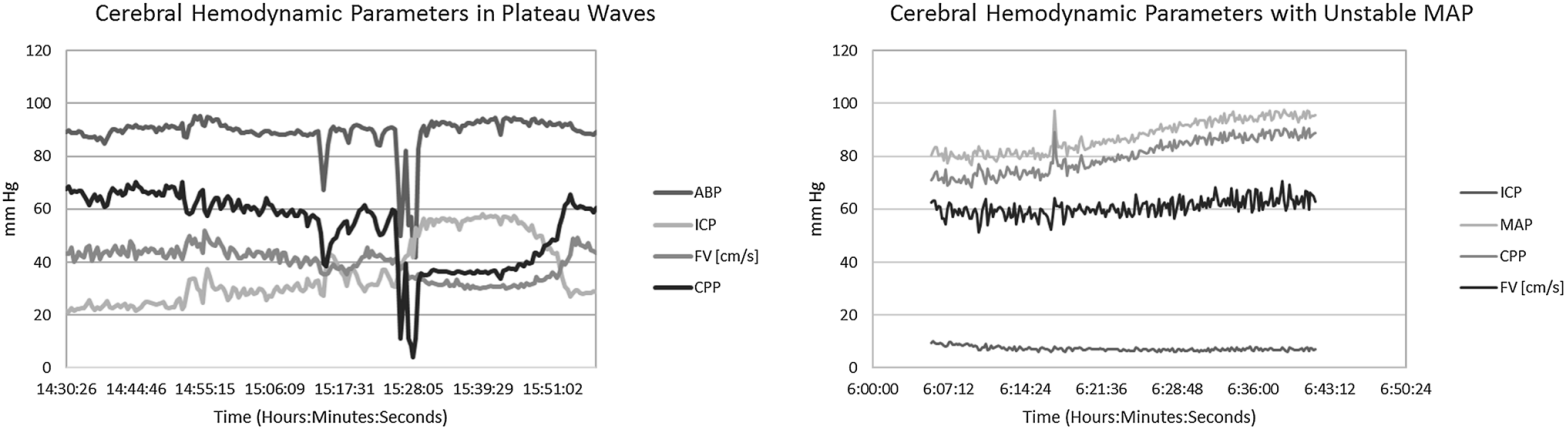
ICP, CPP, and MAP recordings in both plateau wave and unstable MAP patients. *ABP* arterial blood pressure, *CPP* cerebral perfusion pressure, *FV* flow velocity, *ICP* intracranial pressure, *MAP* mean arterial pressure, *mm Hg* millimeters of mercury

A total of 11 patients were eligible for inclusion within the plateau wave cohort of this study, with a total of 18 plateau waves recorded. A total of 9 patients composed the unstable MAP cohort, with 13 separate recordings of unstable blood pressure. Figure 1 displays an example of the ICP, CPP, and MAP recordings from individual patients during plateau waves (Fig. 1a) and unstable blood pressure (Fig. 1b). All available demographic details are listed in Table 1.

**Table 1:** Plateau wave and unstable MAP patient demographics

Patient cohort	Number of patients	Mean age (years)	Male: female ratio	Median admission GCS	Glasgow outcome scale at discharge	
Plateau wave	11	27.2 (range: 17–76)	8:3	5 (range: 3–10)	GOS	# of patients
					Dead	2
					PVS	0
					Severe disability	5
					Moderate disability	4
					Good	0
Unstable MAP	9	25.1 (range: 17–60)	5:4	5 (range: 3–7)	GOS	# of patients
					Dead	2
					PVS	1
					Severe disability	5
					Moderate disability	1
					Good	0

GOS utilized within this study is an inverted GOS, with 5 = death and 1 = good outcome

*GCS* Glasgow Coma Scale, *GOS* Glasgow Outcome Scale*, #* number, *MAP* mean arterial pressure, *PVS* persistent vegetative state

Table 2 summarizes the mean ICP, ABP, CPP, HR, FV, and sPI for both the plateau wave and unstable MAP cohorts. Data for the plateau wave cohort were split into measurements before the plateau wave (i.e., “baseline”) and during the plateau wave, with comparison done via two-tailed *t* test. Data for the unstable MAP cohort were split into the recorded variables during the “Lowest 10%” and “Highest 10%” of recorded arterial blood pressures, with comparison done via two-tailed *t* test.

**Table 2:** Measured and derived signals in plateau and unstable MAP cohorts

	Plateau wave recordings					Unstable MAP recordings				
	Baseline		Plateau			Lowest 10% of MAP		Highest 10% of MAP		
	Mean	SD	Mean	SD	*p* value	Mean	SD	Mean	SD	*p* value
MAP (mm Hg)	96.93	10.12	95.06	8.39	0.52	71.96	15.96	103.65	20.05	0.0002
A1 (mm Hg)	16.41	2.32	15.96	2.25	0.53	15.61	3.76	19.10	5.30	0.07
ICP (mm Hg)	25.60	5.92	50.12	8.66	<0.0001	21.8	10.58	20.65	10.64	0.78
AMP (mm Hg)	2.23	0.73	6.41	1.64	<0.0001	2.51	2.16	1.71	1.15	0.25
CPP (mm Hg)	71.34	12.73	44.94	10.29	<0.0001	50.16	14.91	83.00	19.77	<0.0001
sPI (a.u.)	0.29	0.16	0.48	0.23	0.004	0.51	0.27	0.28	0.12	0.01

*MAP* mean arterial pressure, *CPP* cerebral perfusion pressure, *ICP* intra-cranial pressure, *AMP* fundamental amplitude of ICP, *PI* pulsatility index, *mm Hg* millimeter of Mercury, *SD* standard deviation, *A*1 fundamental amplitude of arterial blood pressure

### Relationships Between CPP, AMP, and sPI During Plateau Waves and Unstable MAP

Linear regression techniques failed to yield satisfactory relationships between CPP and AMP, or CPP and sPI. Their correlation coefficients were poor, and variance measures had large mean squared errors. As the scatterplots for each of these comparisons produced a nonlinear pattern, we utilized nonlinear regression analyses (with functions within XLSTAT and IBM SPSS Statistics 23 software) to determine the relationships displayed between these variables during ICP plateau waves, using an inverse function that we have previously theorized to characterize this relationship. Nonlinear regression analysis for CPP versus sPI in each individual plateau wave patient is shown in Appendix A of the Supplementary Materials. Nonlinear regression analysis for CPP versus sPI in each unstable MAP patient is shown in Appendix B of the Supplementary Materials.

**Fig. 2:**
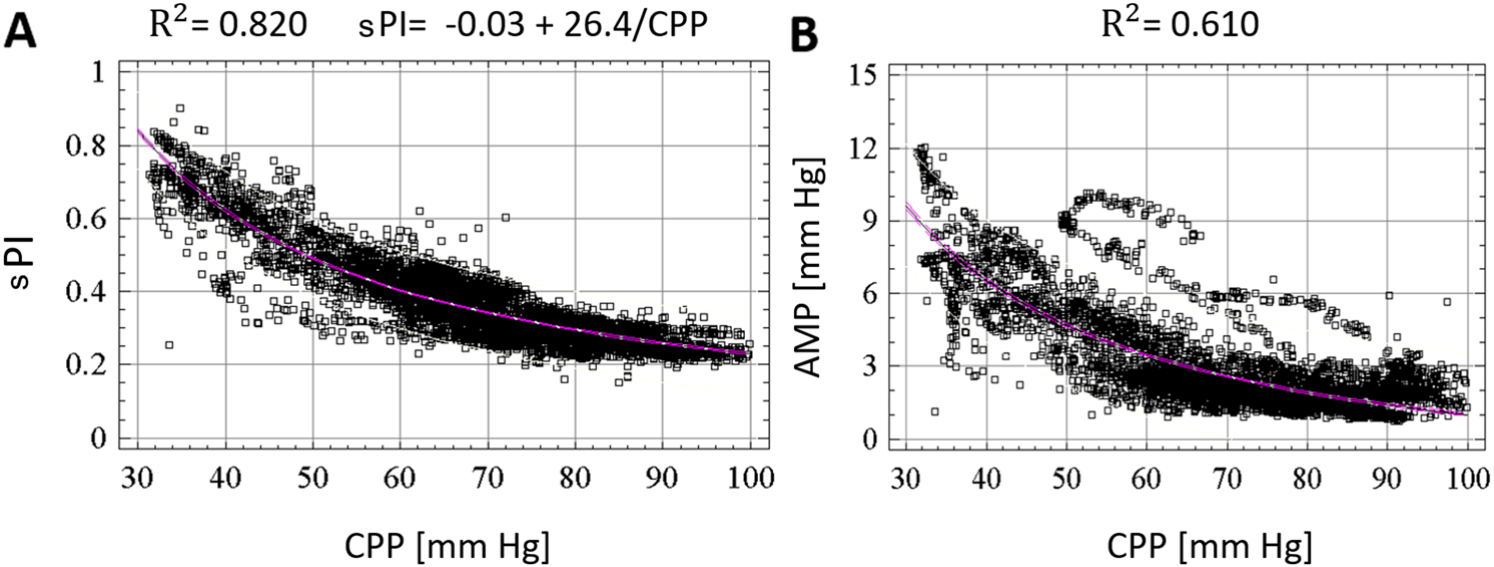
Nonlinear regression analysis of CPP versus sPI (F1/FV) and CPP versus AMP in plateau cohort. **a** Nonlinear regression of CPP versus sPI. **b** Nonlinear regression of CPP versus AMP. *AMP* ICP pulse amplitude, *CPP* cerebral perfusion pressure, *F1* amplitude of fundamental frequency of FV, *FV* mean blood flow velocity in the mean cerebral artery (MCA), *mm Hg* millimeter of mercury, *sPI* spectral pulsatility index

**Fig. 3:**
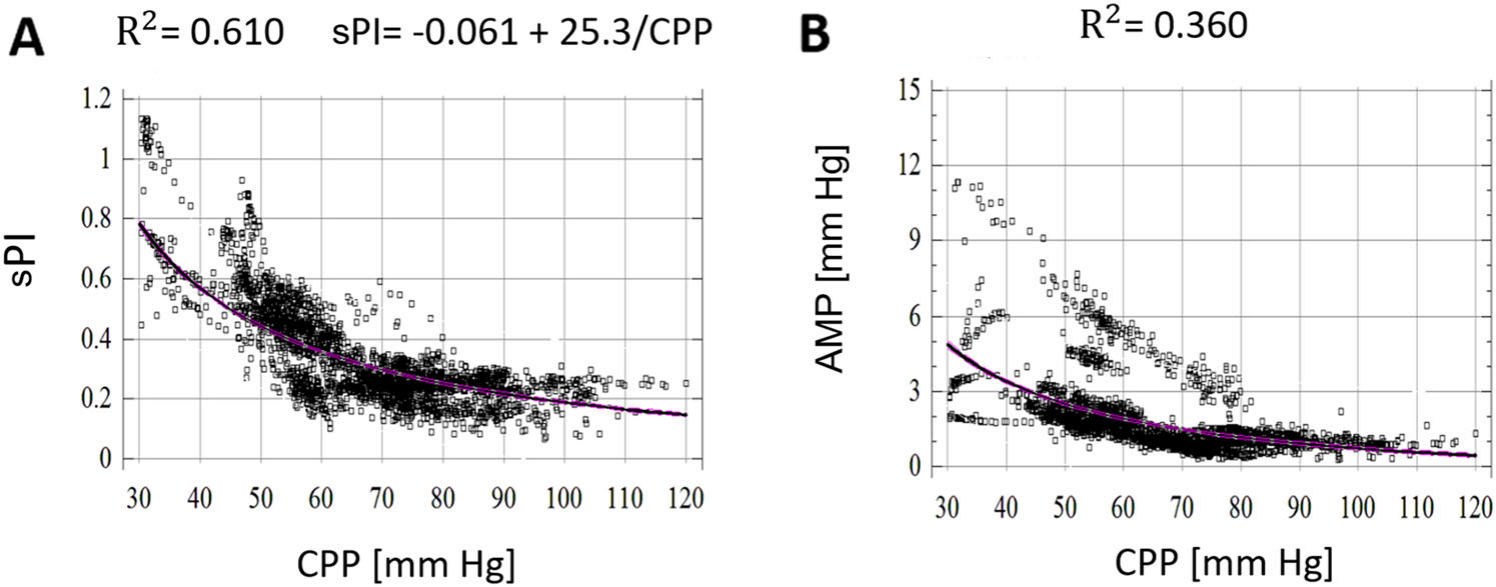
Nonlinear regression analysis of CPP versus sPI (F1/FV) and CPP versus AMP in unstable MAP cohort. **a** Nonlinear regression of CPP versus sPI. **b** Nonlinear regression of CPP versus AMP. *AMP* ICP pulse amplitude, *CPP* cerebral perfusion pressure, *F1* amplitude of fundamental frequency of FV, *FV* mean blood flow velocity in the mean cerebral artery (MCA), *mm Hg* millimeter of mercury, *sPI* spectral pulsatility index

The results of both the nonlinear regression across the compiled plateau wave patient data for CPP versus sPI are shown in Fig. 2a. Similarly, the nonlinear regression for CPP versus AMP is shown for Fig. 2b. The corresponding results for the compiled unstable MAP patient data are shown in Fig. 3a and Fig. 3b, respectively.

#### CPP versus AMP

Nonlinear regression analysis of the relationship between CPP and AMP in plateau wave patients produced an inverse relationship between CPP and AMP (*R* ^2^ = 0.610). Nonlinear regression analysis of the relationship between CPP and AMP in unstable MAP patients produced an inverse relationship between the two parameters (*R* ^2^ = 0.36).

#### CPP versus sPI

Similarly, nonlinear regression analysis of the relationship between CPP and sPI in the plateau wave cohort produced an inverse relationship (*R* ^2^ = 0.820), best described by the following function:

$${\text{sPI = }}a{ + }\left( {b / {\text{CPP}}} \right)$$

with CPP measured in mm Hg, and the statistical analysis concluding: *a* = −0.03 and *b* = 26.4. When the individual plateau wave patients were analyzed via nonlinear regression, the mean and standard deviation for the values of “*a*” and “*b*” were: *a* = 0.005 ± 0.061, *b* = 23.61 ± 6.33.

Similarly, nonlinear regression analysis of CPP versus sPI in the unstable MAP cohort demonstrated an inverse relationship between CPP and sPI (*R* ^2^ = 0.61), as shown in Fig. 3a. As seen within the plateau cohort’s nonlinear regression of CPP versus sPI, the model of best fit was the same as in plateau waves, showing the same function (with CPP measured in mm Hg, *a* = −0.061 and *b* = 25.3). When the individual unstable MAP patients were analyzed via nonlinear regression, the mean and standard deviation for the values of “*a*” and “*b*” were: *a* = −0.144 ± 0.391, *b* = 27.43 ± 21.72. Interestingly, both relationships closely resemble and support the inverse nonlinear relationship between CPP and PI previously proposed by de Riva et al. [4].

The “*a*” and “*b*” values calculated for each patient cohort were compared in a two-tailed independent-samples *t* test to evaluate significant differences between the plateau wave versus unstable MAP cohorts. Levene’s test for equality of variances was assumed and dictated a nonsignificant difference between both the “*a*” and the “*b*” values obtained from the two groups (*t*[27] = −1.507, *p* = 0.143 and *t*[27] = 0.670, *p* = 0.509, respectively).

The effects of this hypothesis were further examined to determine whether each group’s sets of “a” values were statistically different from the test value of 0 via two-tailed one-sample *t* tests. There was a nonsignificant difference between 0 and the “*a*” values in unstable MAP patients as well as in plateau wave patients (*t*[12] = −1.330, *p* = 0.208 and *t*[15] = 0.300, *p* = 0.768, respectively).

#### Relationships Between ICP, AMP, and sPI During Plateau Waves and Unstable MAP

Unlike the case for relationships between CPP versus sPI and AMP (where nonlinear relationships were found), linear regression techniques yielded robust relationships of ICP with calculated variables in the plateau patient cohort.

**Fig. 4:**
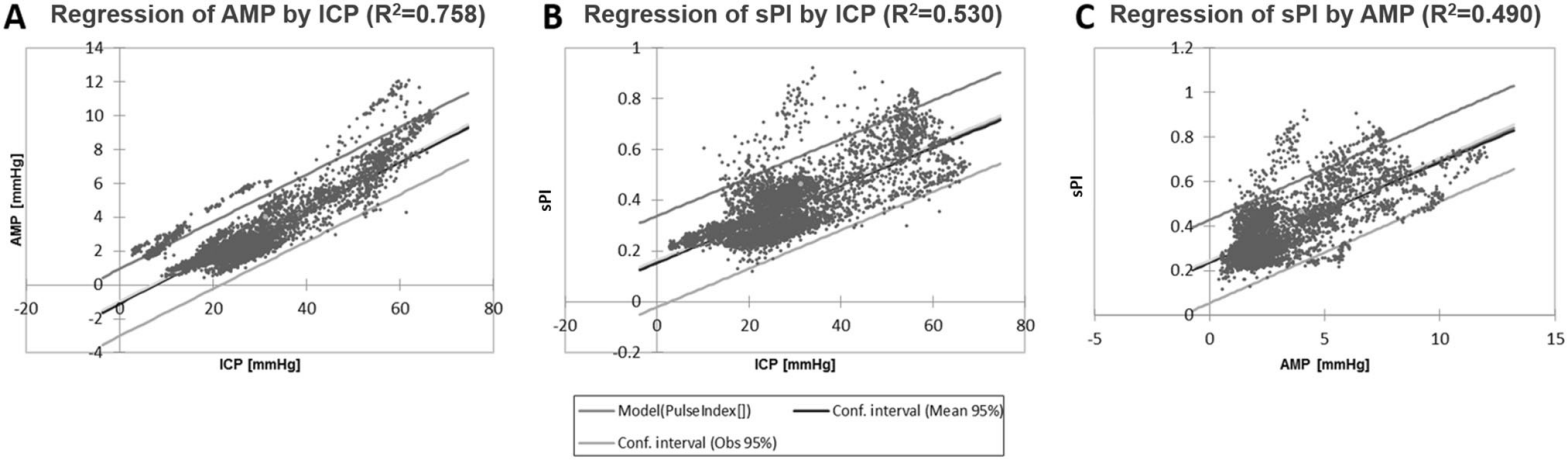
Linear regression analysis of ICP versus AMP, ICP versus sPI, and AMP versus sPI in plateau cohort. **a** Linear regression of ICP versus AMP. **b** Linear regression of ICP versus sPI. **c** Linear regression of AMP versus sPI. *AMP* ICP pulse amplitude, *ICP* intracranial pressure, *mm Hg* millimeters of mercury, *sPI* spectral pulsatility index, *R*2 coefficient of determination

The relationship between ICP and AMP across the compiled patient data for the plateau wave cohort is shown in Fig. 4a. A statistically significant linear relationship was described between ICP and AMP (*r* = 0.871, *R* ^2^ = 0.758). Similarly, a statistically significant linear relationship was described between ICP and sPI (*r* = 0.728, *R* ^2^ = 0.530), as displayed in Fig. 4b. The relationship between AMP and sPI is displayed in Fig. 4c. Linear regression techniques yielded a significant relationship between AMP and sPI (*r* = 0.700, *R* ^2^ = 0.490).

**Fig. 5:**
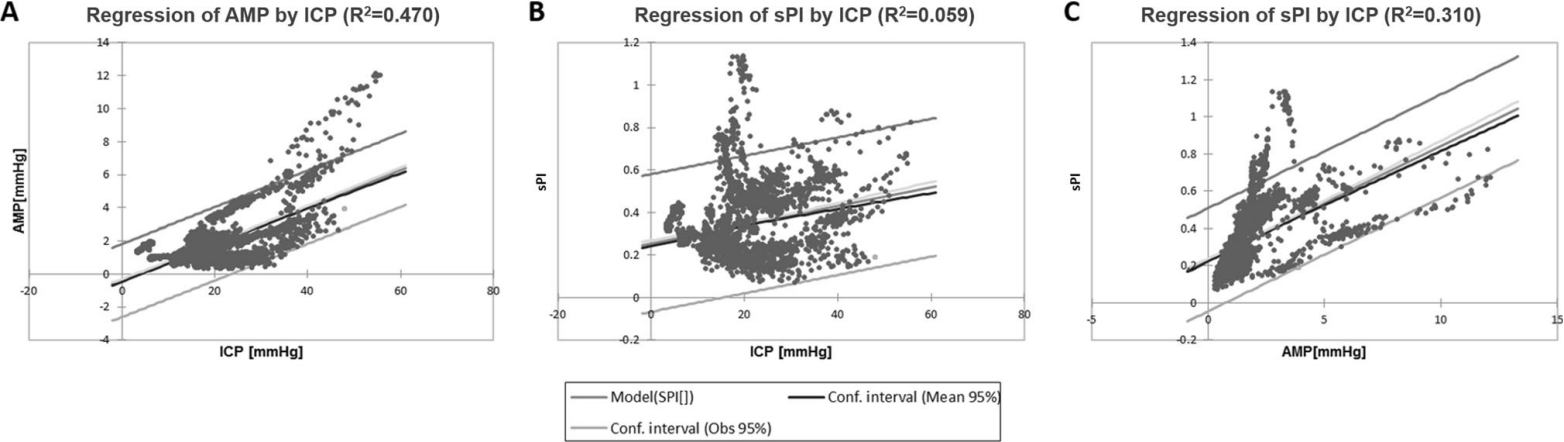
Linear regression analysis of ICP versus AMP, ICP versus sPI, and AMP versus sPI in unstable MAP cohort. **a** Linear regression of ICP versus AMP. **b** Linear regression of ICP versus sPI. **c** Linear regression of AMP versus sPI. *AMP* ICP pulse amplitude, *ICP* intracranial pressure, *mm Hg* millimeters of mercury, *sPI* spectral pulsatility index, *R*2 coefficient of determination

While linear regression also demonstrated significant relationships between ICP and AMP across the unstable MAP cohort, these relationships were less robust (Fig. 5). A statistically significant linear relationship was described between ICP and AMP (*R* ^2^ = 0.470). A very weak linear relationship was described between ICP and sPI (*R* ^2^ = 0.059), as displayed in Fig. 5b. Finally, the relationship between AMP and sPI was linear (*R* ^2^ = 0.310) (Fig. 5c).

## Discussion

In the past, observations of brain pulsatility in the scenario of lowering CPP [10] and increasing ICP [11] were reported, although much mixed methodology was used in those works. In this study, we used a unified method to compare the same relationship in clinical conditions where CPP is affected either by increasing ICP or by the oscillations of unstable MAP.

Through the application of linear and nonlinear regression analysis, we have displayed both confirmatory and new results regarding the relationships between TCD-based PI and invasively-measured cerebral hemodynamic indices, ICP and CPP. This is “old” data harvested from the “Cambridge database” of high-resolution recorded signals from the 1990s, as neuro-intensive care TBI patients at that time were not treated according to rigorous CPP-/ICP-oriented protocol—therefore, incidences of lowering CPP were recorded more easily. This is a relevant major aspect of these data recordings given that it is uncommon to have high-resolution datasets in the absence of CPP-directed therapy post-TBI.

Here, we have demonstrated that large fluctuations in CPP, either via changes in ICP or MAP, hold true the inverse nonlinear relationship between CPP versus sPI, and this relationship can be best described through the function: PI = *a* + (*b*/CPP); with a ~ 0 (i.e., plateau patients, *a* = −0.03; unstable MAP, *a* = −0.06) and b almost identical between both cohorts (i.e., plateau patients, *b* = 26.4; unstable MAP, *b* = 25.3). Furthermore, nonlinear regression analysis of each individual patient in both cohorts shows that the value for “*a*” is also close to 0. This was displayed strongly within the plateau wave cohort (mean “*a*” = 0.005; SD = 0.061). The unstable MAP cohort displayed this same relationship, but less substantially (mean “*a*” = −0.144; SD = 0.391). The statement that “*a*” was no different from 0 was further solidified via *t* test analysis demonstrating no statistically significant difference between “*a*” and 0 in both cohorts. Therefore, if “*a*” is essentially equal to 0, then the relationship between CPP versus sPI can be approximated by the relation: PI = *b*/CPP, with *b* ~ 25. This closely models the relation proposed by de Riva et al. [6] and provides the first evidence in support of this mathematical relation between CPP and PI in human models.

Secondly, we have also demonstrated the positive linear correlations between ICP versus AMP, ICP versus sPI, and AMP versus sPI in both the plateau wave and unstable MAP cohorts. Linear regression analysis of ICP versus AMP displayed the most robust linear relationship. Although the relationship between ICP versus nonspectral methods of PI calculation had been already described [12–15], limited literature exists by utilizing spectral methods for PI determination. Furthermore, the relationship between ICP versus AMP and AMP versus sPI is seldom described, leaving our manuscript as a nice and clear example of their linear relationships.

Third, it is also remarkable that the relationship between CPP and AMP also followed an inverse nonlinear relationship through nonlinear regression techniques. Again, this was also confirmed for both plateau wave and unstable MAP cohorts.

On the other hand, ICP seems to have a stronger link to intra-cranial/extra-vascular parameters (i.e., AMP, with an *R* ^2^ = 0.758) compared to intra-vascular measurements (i.e., sPI, with an *R* ^2^ = 0.530). Conversely, we could show that CPP displays a stronger relationship to intra-vascular parameters (i.e., sPI, with an *R* ^2^ = 0.820) versus extra-vascular intra-cranial measures (i.e., AMP, with an *R* ^2^ = 0.610).

As a last point, the fact that sPI is a smooth inverse function of CPP makes it very difficult to prove that the CPP level below which sPI starts to increase could denote the lower limit of autoregulation. This would mean that the brain is on the verge of becoming unable to maintain a constant level of blood flow. This thesis was proposed in the past [10], but later experimental challenges have proven it wrong [16].

### Clinical Implications

The most recent edition of the Brain Trauma Foundation Guidelines recommends that CPP be directed towards the target range of 60–70 mm Hg. Constraining CPP between these values is thought to prevent either the hyper- or hypo-perfusion that could, respectively, increase patient risk of poor outcome. When considering trends across individual patient data, all sPI versus CPP curves suggest that values of sPI around 0.4 correspond to CPP values around 60 mm Hg. In this manner, sPI can easily be interpreted by clinicians as an indicator of the accepted “safe” lower bound of CPP [17]. Furthermore, through the above analysis we have been able to demonstrate the correlation between TCD-based sPI and CPP. This reinforces previous literature stating that TCD potentially provides the ability for a noninvasive estimation of CPP. Finally, we were able to demonstrate that the relationship between CPP- and TCD-based sPI is maintained during extremes of physiology (i.e., plateau waves and unstable MAP). Thus, if the clinician is to apply this methodology of noninvasive CPP estimation, our data suggest that the relationship between sPI and CPP should hold true, regardless of the individual clinical situation and extremes of physiology seen at the time of measurement.

### Limitations

Our study has several limitations that need to be acknowledged. First, our analyses are based on observational data, rather than a prospective recording of response to a change in CPP. Consequently, many confounders may have affected critical variables, and the data access we have (and the relatively small volume of data compatible with ICM+ during this period) does not allow us to fully account for these. Second, our results are derived from only 11 sets of patient data containing 18 distinct plateau waves and nine datasets containing 13 instances of variable MAP. Consequently, extrapolation of this data to all patients with TBI is not possible, and confirmation of the described relationships will need to occur through comparative analysis of larger datasets.

Third, our nonlinear regression techniques for the relationships between CPP versus AMP and CPP versus sPI described the best fit with an inverse nonlinear function. However, with a total of only 20 patients, larger datasets are needed to better delineate and further prove this inverse relationship. Given that our patient population was so small, the next step is to validate our findings within a large TBI cohort to show that the proposed relationship holds. The relation yielded via nonlinear regression cannot be extrapolated and must serve only as a point of interest in the relationship between CPP versus AMP and CPP versus sPI, providing preliminary supporting evidence for the theorized nonlinear relation previously described in the literature [6]. Fourth, within the unstable MAP cohort, it is difficult clinically to isolate pure MAP from pure ICP contributions to changes in CPP. These patients exhibit significant fluctuations in various physiologic measures, as it is shown in Table 2. Finally, patients with severe TBI and plateau waves are an extreme cohort of critically ill patients, with injuries that may yield abnormal physiologic brain properties. Therefore, the relationships described in this small study cannot necessarily be applied to all TBI patients.

## Conclusions

In severe TBI patients with plateau waves or unstable MAP, the relationships between CPP and pulsatility of brain signals are inversely proportional, no matter the mechanism that lowers CPP. ICP versus AMP, ICP versus sPI, and AMP versus sPI display positive linear correlations.

## Electronic supplementary material

Below is the link to the electronic supplementary material.

Supplementary material 1 (DOC 492 kb)

Supplementary material 2 (DOC 448 kb)
