# Supplementary material for: Relationship Between Brain Pulsatility and Cerebral Perfusion Pressure: Replicated Validation Using Different Drivers of CPP Change
Source: Neurocrit Care. 2017 May 25;27(3):392–400. doi: 10.1007/s12028-017-0404-9 (PMC5700211; doi:10.1007/s12028-017-0404-9)
Supplement: Supplementary file 1 — Supplementary material 1 (DOC 492 kb) [file 12028_2017_404_MOESM1_ESM.doc]

**Appendix A – Non-linear Regression Between CPP and PI for Individual Patients with Plateau Waves**

***Note:**

**x-axis = CPP measured in mm Hg**

**y-axis = PI (F1/FV); no unit**

**Coefficients of Determination:**

| **P1 = 0.93** | **P2 = 0.94** |
| --- | --- |
| **P3 = 0.75** | **P4 = 0.81** |
| **P5 = 0.97** | **P6 = 0.80** |
| **P7 = 0.87** | **P8 = 0.92** |
| **P9 = 0.83** | **P10 = 0.89** |
| **P11 = 0.84** | **P12 = 0.73** |
| **P13 = 0.56** | **P14 = 0.92** |
| **P15 = 0.94** | **P16 = 0.98** |

**
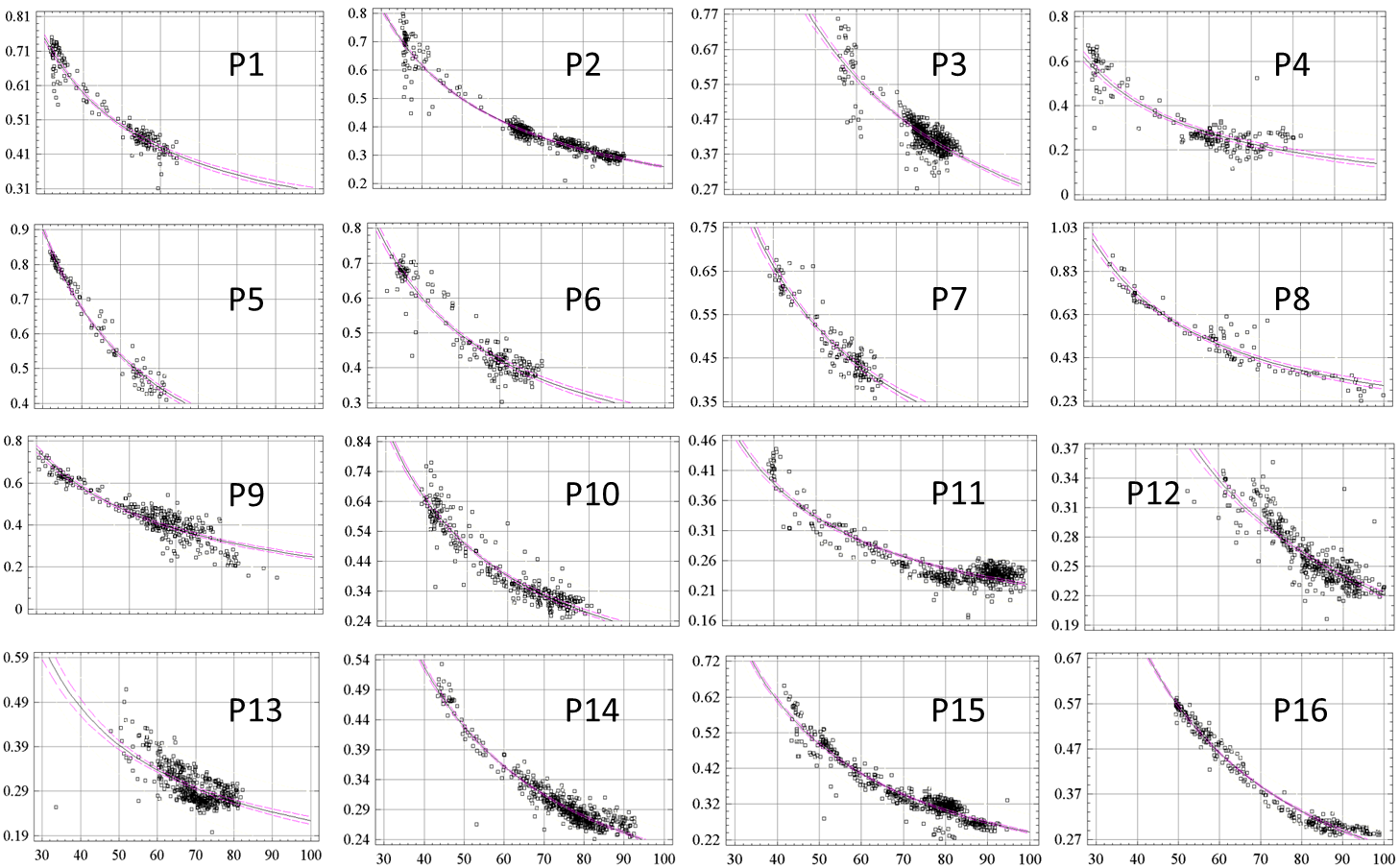
**
