# Supplementary material for: Relationship Between Brain Pulsatility and Cerebral Perfusion Pressure: Replicated Validation Using Different Drivers of CPP Change
Source: Neurocrit Care. 2017 May 25;27(3):392–400. doi: 10.1007/s12028-017-0404-9 (PMC5700211; doi:10.1007/s12028-017-0404-9)

**Appendix B – Non-linear Regression Between CPP and PI for Individual Patients with Unstable MAP**

***Note: x-axis = CPP measured in mm Hg, y-axis = PI (F1/FV); no unit**


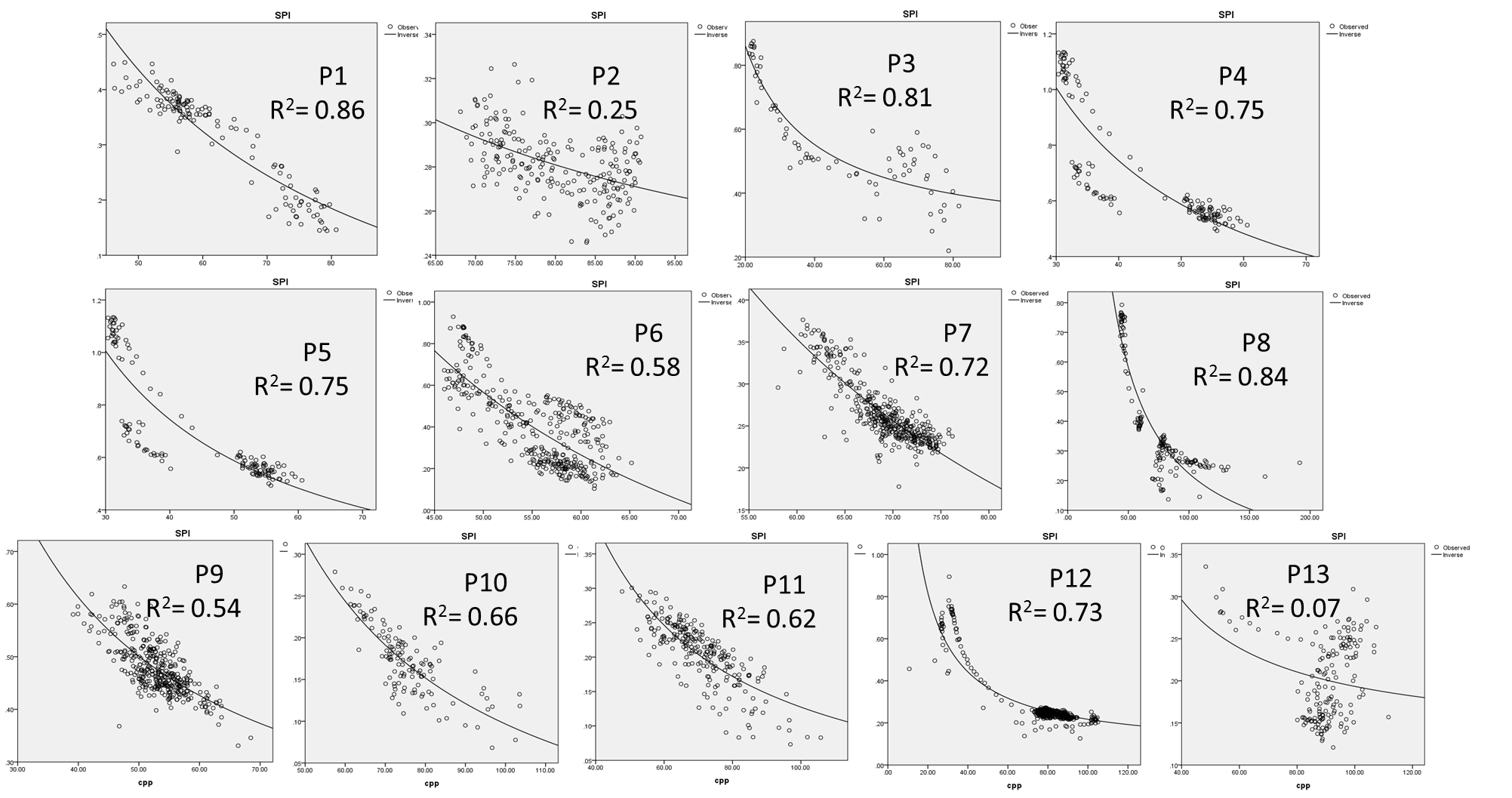

Supplement: Supplementary file 2 — Supplementary material 2 (DOC 448 kb) [file 12028_2017_404_MOESM2_ESM.doc]
